# Transforming growth factors β and their signaling pathway in renal cell carcinoma and peritumoral space—transcriptome analysis

**DOI:** 10.1007/s12094-023-03350-y

**Published:** 2023-12-12

**Authors:** Dariusz Kajdaniuk, Dorota Hudy, Joanna Katarzyna Strzelczyk, Krystyna Młynarek, Szymon Słomian, Andrzej Potyka, Ewa Szymonik, Janusz Strzelczyk, Wanda Foltyn, Beata Kos-Kudła, Bogdan Marek

**Affiliations:** 1 Department of Pathophysiology, Chair of Pathophysiology and Endocrinology, Faculty of Medical Sciences in Zabrze, Medical University of Silesia, H. Jordana 19, Zabrze, 41-808 Katowice, Poland; 2 Department of Medical and Molecular Biology, Faculty of Medical Sciences in Zabrze, Medical University of Silesia, Katowice, Poland; 3 Department of Urology, Regional Specialist Hospital No. 3, Rybnik, Poland; 4 Department of Anesthesiology and Intensive Care, Brothers Hospitallers of Saint John of God Hospital in Katowice, Katowice, Poland; 5 Department of Endocrinology and Neuroendocrine Tumors, Chair of Pathophysiology and Endocrinology, Faculty of Medical Sciences in Zabrze, Medical University of Silesia, Katowice, Poland

**Keywords:** Transforming growth factor beta, TGF beta, Smad, Renal cell carcinoma, Tumor microenvironment, Cancer immunology

## Abstract

**Purpose:**

The aim of the study was to verify hypotheses: Are transforming growth factors TGFβ1-3, their receptors TGFβI-III, and intracellular messenger proteins Smad1-7 involved in the pathogenesis of kidney cancer? What is the expression of genes of the TGFβ/Smads pathway in renal cell carcinoma (RCC) tissues, peritumoral tissues (TME; tumor microenvironment), and in normal kidney (NK) tissue?.

**Methods:**

Twenty patients with RCC who underwent total nephrectomy were included into the molecular analysis. The mRNA expression of the genes was quantified by RT-qPCR.

**Results:**

The study showed that the expression of the genes of TGFβ/Smads pathway is dysregulated in both RCC and the TME: TGFβ1, TGFβ3 expression is increased in the TME in comparison to the NK tissues; TGFβ2, TGFβ3, TGFβRI, TGFβRIII, Smad1, Smad2, Smad3, and Smad6 are underexpressed in RCC comparing to the TME tissues; TGFβRI, TGFβRIII, and Smad2 are underexpressed in RCC in comparison to the NK tissues.

**Conclusion:**

On the one hand, the underexpression of the TGFβ signaling pathway genes within the malignant tumor may result in the loss of the antiproliferative and pro-apoptotic activity of this cytokine. On the other hand, the overexpression of the TGFβ/Smads pathway genes in the TME than in tumor or NK tissues most probably results in an immunosuppressive effect in the space surrounding the tumor and may have an antiproliferative and pro-apoptotic effect on non-neoplastic cells present in the TME. The functional and morphological consistency of this area may determine the aggressiveness of the tumor and the time in which the neoplastic process will spread.

**Supplementary Information:**

The online version contains supplementary material available at 10.1007/s12094-023-03350-y.

## Introduction

Transforming growth factor β1 (TGFβ1) is a protein that regulates cell proliferation, growth, differentiation, and movement. It acts as an apoptosis regulator in normal and pathologically altered tissues, and thus controls the balance between replication and cell death [1]. TGFβ1 also plays an important role in maintaining immune homeostasis [2] due to its immunomodulatory effect [1, 3–6], its role as a regulator of immune tolerance [2], and its involvement in suppression of the immune response. It inhibits proliferation, differentiation and activity of the cells participating in the humoral and cellular response, reduces expression of MHC molecules, inhibits secretion of cytokines, reduces production of antibodies, and exerts an inhibitory effect on some of the functions of the NK cells, e.g., on their cytotoxic activity [1, 3–6]. A subtype of CD4 + T cells, regulatory T cells (Treg), are the major producer of the latent TGFβ1. At the same time, TGFβ1 signaling is crucial for Treg development. The active TGFβ1 also modulates the differentiation of other T cell subsets [2]. It prevents Th1 and Th2 differentiation by suppressing Signal Transducer, Activator of Transcription (STAT4) and GATA-3 expression, allowing development of Th17 cells [7–9]. Differentiation of naive T cells towards the Th17 phenotype is supported by several “differentiating cytokines” including TGFβ [10, 11]. In addition, being one of the key mediators of fibrogenesis, TGFβ1 is important for scarring and tissue reconstruction processes [1, 4, 5, 12]. The functions of TGFβ2 and TGFβ3 are still poorly understood. They probably regulate the cell proliferation, growth, differentiation and movement, participate in tissue remodeling, wound healing and tumor formation [1]. TGFβ2 can also induce apoptosis [13].

TGFβ1-3 acts through transmembrane TGFβ type I-III receptors (TGFβRI-III) [1, 14], and the Smad signal transducer system [1, 15, 16]. After being activated by phosphorylation, the receptor complex transmits the signal onto intracellular proteins Smad2 and Smad3, the so-called R-Smad (Receptor-associated Smad). As a result, R-Smad dissociates from TGFβ-receptor and forms a complex with Co-Smad (Common partner Smad) i.e., Smad4. The newly formed complex is then transported into cell nucleus, where it regulates the transcription of TGFβ-dependent genes. In this light, R-Smad are transcription factors for specific genes [1, 2, 17, 18], while Smad4, being a target for other transcription factors, also participate in the regulation of TGFβ-dependent gene expression. The above-mentioned signal transduction can be inhibited by Smad6 and Smad7, the so-called I-Smads (Inhibitory Smads). Smad7 forms a stable complex with activated TGFβRI, thereby impairing the R-Smad phosphorylation. This, in result, inhibits the whole signal cascade. At the same time, while Smad7 diminishes cell response to TGFβ, its expression is induced by this factor, in a mechanism of an autoregulatory negative feedback loop [1, 15, 19]. In a disease state, Smads can also interact with other signaling pathways. Smad-independent TGFβ signaling occurs through molecular pathways such as the mitogen-activated protein kinase and nuclear factor-κB pathways, c-Jun N-terminal kinase (JNK) and p38 mitogen-activated protein kinases (MAPKs) pathway, phosphoinositide 3-kinase (PI3K) and protein kinase B (PKB) pathway, and small Rho-like GTPase signaling pathway [20–23].

In early stages of tumor development, tumor cells respond to the antimitotic and pro-apoptotic effects of TGFβ1. Thus, initially, TGFβ1 plays a role of a tumor suppressor [1, 5, 14, 21]. In later stages TGFβ1 acts as a tumor promoter by modulating genomic instability, angiogenesis, lymphangiogenesis, immune suppression, immune evasion, epithelial-mesenchymal transition, endothelial-mesenchymal transition, and cell motility [1, 5, 14, 17, 21, 23]. It creates tumor microenvironment (TME) favorable to the tumor growth and metastasis, subsequently increasing the invasiveness of the cancer cells [1, 5, 14, 21, 24].

The most common type of kidney cancer (KC) in adults is renal cell carcinoma (RCC) (approximately 90% of all kidney malignancies) [25, 26]. RCC can be treated with partial or radical nephrectomy with a favorable outcome [25]. At the time of preliminary diagnosis, 20%–30% of patients with RCC have local or distant metastases [27].

The Cancer Genome Atlas Project (TCGA) (https://www.cancer.gov/tcga) provides a source of primary cancer samples collected from 11,014 patients. Within the group 20,901 genes, 2,108,204 mutations were detected, characterized, and analyzed. Among 943 patients with RCC, 44,544 mutations in 19,786 genes were detected. In the primary RCC tumor cases, the most commonly affected genes were, by frequency, VHL (von Hippel-Lindau tumor suppressor), PBRM1 (polybromo 1), TTN (titin), MUC16 (mucin 16, cell surface associated), SETD2 (SET domain containing 2, histone lysine methyltransferase), BAP1 (BRCA1 associated protein 1), LRP2 (LDL receptor related protein 2), DST (dystonin), KMT2C (lysine methyltransferase 2C), and TP53 (tumor protein p53) [28].

The aim of the study was to verify following research hypotheses: Are transforming growth factors TGFβ1-3, their receptors TGFβI-III, and intracellular messenger proteins Smad1-7 involved in the pathogenesis of kidney cancer? What is the expression (mRNA) of genes of the TGFβ/Smads pathway in renal cell carcinoma (RCC) tissues, the tissues surrounding the tumor (TME; tumor microenvironment), and in the normal kidney (NK) tissue?

## Materials and methods

### Patients

This study was performed in line with the principles of the Declaration of Helsinki. Approval was granted by the Bioethical Committee of the Medical University of Silesia (No. PCN/0022/KB1/118/19). Informed consent was obtained from all individual participants included in the study. Thirty-three patients with a newly diagnosed kidney tumor who needed surgical treatment due to suspected kidney cancer (KC) were recruited for the study. The study inclusion criteria were age over 18 and under 75 years, as well as the diagnosis of kidney cancer based on a clinical picture and confirmed by typical imaging tests (such as CT, MRI, ultrasound, angiography). Exclusion criteria involved age under 18 and over 75 years, presence of severe comorbidities like renal failure (eGFR < 45 ml/min./1.73m^2^), liver failure (bilirubin > 34.2 µmol/l), symptomatic circulatory insufficiency, symptomatic respiratory failure, severe neurological diseases, and mental disorders. Four patients with histopathological diagnosis of urothelial carcinoma were excluded from the study. Nine patients with histopathological diagnosis of RCC (7 ccRCC; 2 papillary RCC) who underwent partial nephrectomy i.e., nephron sparing surgery (NSS) were also excluded from this analysis.

Finally, the study group consisted of 20 patients with histopathological diagnosis of renal cell carcinoma (RCC) who underwent total nephrectomy. The RCC group included patients with ccRCC (16), papillary RCC (2), chromophobe RCC (1), and sarcomatoid RCC (1). The RCC group comprised 13 men and 7 women aged 64.65 ± 9.59 (mean ± SD) years old (median 65; Q_L_-Q_U_ 60.5–71). Kidney tissues removed during the surgical treatment, taken from the tumor, from the immediate space surrounding the tumor, and from a site distant from the tumor assessed as normal, were secured in special tubes (fixRNA; EURx, Poland), and then stored at -75 °C until molecular biology analysis.

### Methods

In the study group, mRNA expression of the genes encoding the growth factors TGFβ1, TGFβ2, TGFβ3, their TGFβRI, TGFβRII, TGFβRIII receptors and the intracellular messenger proteins Smad1, Smad2, Smad3, Smad4, Smad5, Smad6, Smad7 were analyzed using RT-qPCR (Reverse Transcriptase quantitative Polymerase Chain Reaction). Molecular analysis was performed in the RCC tissues, the tissues surrounding the tumor and in the normal kidney tissue.

Homogenization of tissue was done with FastPrep-24 instrument and Lysing Matrix D (MP Biomedicals, USA, #116,913,050-CF) in lysing buffer from RNA isolation kit. Isolation of RNA was done with RNA isolation kit (Biovendor, Czech Republic, #RIK001) according to producent procedure. RNA concentration was measured with Pearl nanophotometer (Implen, Germany). Probes were stored in − 80 °C until further analysis.

10 ng of total RNA was transcribed into cDNA with High-Capacity cDNA Reverse Transcription Kit with RNase Inhibitor (Applied Biosystems, USA, #4,374,966) on Mastercycler personal (Eppendorf, Germany). 10 ng of RNA (10 µl) was mixed with 2 × RT master mix which consisted of 2 µl 10 × RT buffer, 0.8 µl 25 × dNTP mix (100 mM), 2 µl 10 × RT random primers, 1 µl MultiScribe™ reverse transcriptase, 1 µl RNase inhibitor, 3.2 µl nuclease-free water. Reaction was assessed according to protocol: 10 min in 25 °C, 120 min in 37 °C, 5 min in 85 °C and 4 °C until further analysis. cDNA was stored in − 20 °C.

Expression of genes was measured by PCR done in triplicates on Quant Studio 5 instrument (Applied Biosystems, USA, # A47326) with TaqMan™ Fast Advanced Master Mix (Applied Biosystems, USA, #4,444,964) in 20 µl volume. Reaction mix consisted of 1 µl cDNA, 1 µl primers, 10 µl 2 × master mix and 8 µl nuclease-free water. Genes and assay ID of primers are listed in table (Table 1). Relative expression of genes was calculated from ΔΔCT (http://docs.appliedbiosystems.com/pebiodocs/04303859.pdf) with GAPDH as reference gene and mix of six healthy tissue (far margin) cDNAs as calibrator. Reaction conditions were as follow: hold 20 s in 95 °C than 60 cycles with 1 s in 95 °C and 20 s in 60 °C.

**Table 1:** Genes and primers for the assays used in this study

Gene name	Assay ID
GAPDH	Hs03929097_g1
TGFβ1	Hs00171257_m1
TGFβ2	Hs00234244_m1
TGFβ3	Hs01086000_m1
TGFβRI	Hs00610320_m1
TGFβRII	Hs00234253_m1
TGFβRIII	Hs00234257_m1
Smad1	Hs00195432_m1
Smad2	Hs00183425_m1
Smad3	Hs00969210_m1
Smad4	Hs00929647_m1
Smad5	Hs00195437_m1
Smad6	Hs00178579_m1
Smad7	Hs00178696_m1

### Statistical methods and tools

The Shapiro–Wilk test was used to determine the normality of samples. T test or Mann–Whitney U test and Spearman correlation were used to determine the significant differences and relationships in parameters. A significant level was set at *p* value < 0.05. The data were presented as median with the range. All statistical analyses were performed using STATISTICA 13.3 (StatSoft. Inc., Tulsa, Oklahoma, USA).

## Results

**Fig. 1:**
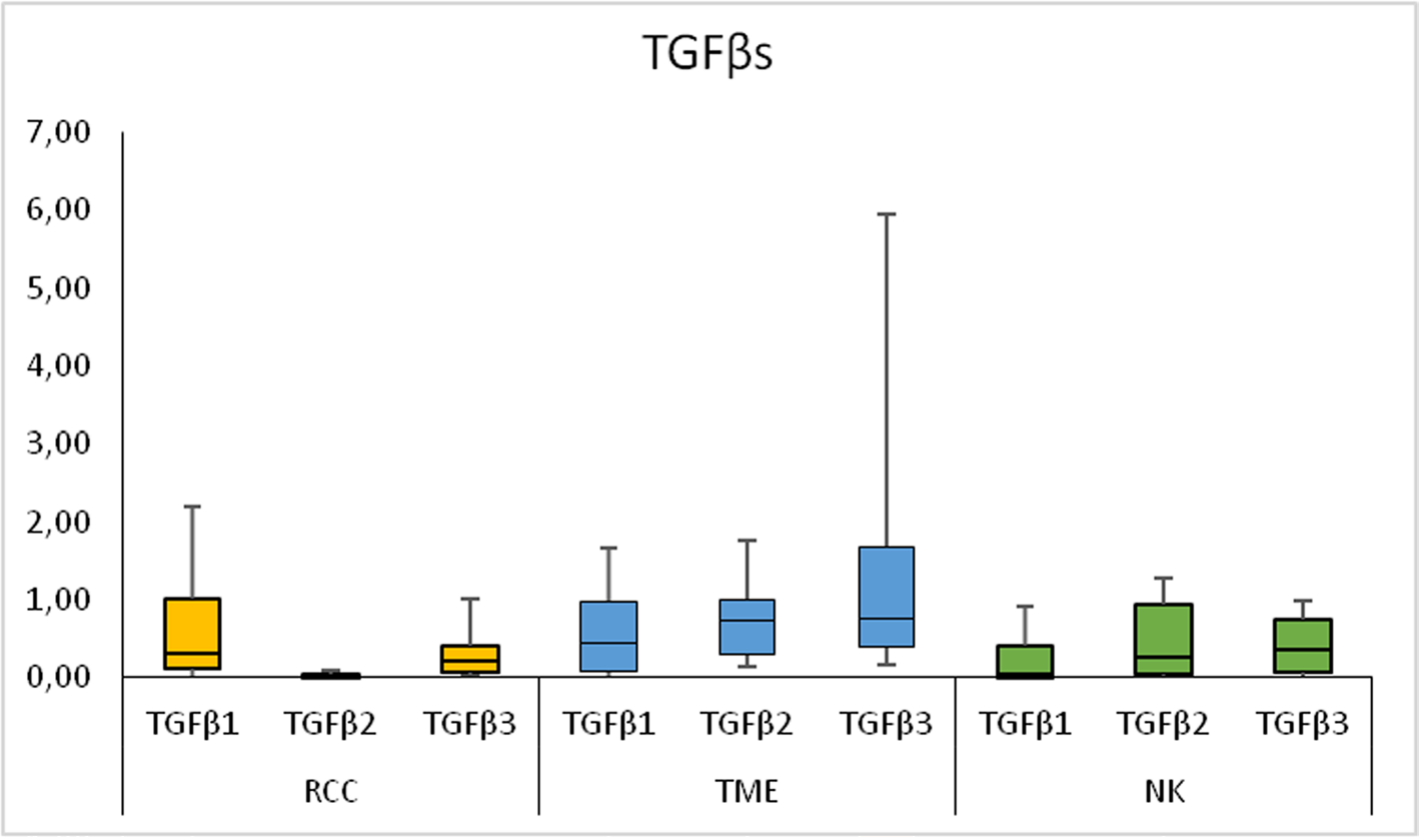
The expression (mRNA) of genes of the transforming growth factors (TGFβs) in renal cell carcinoma (RCC), tumor microenvironment (TME) and in normal kidney (NK) tissues. *RQ* relative quantification, Relative gene expression levels, median, *Q*_*L*_ lower quartile, *Q*_*U*_ upper quartile, lowest value, highest value are presented

According to Table 2: there were no significant differences in the TGFβs-encoding mRNA expression levels between RCC and corresponding normal kidney (NK) tissues. However, the expression of TGFβ1 mRNA in the TME was significantly higher in comparison to the NK tissues. The decreased expression of TGFβ2 mRNA in RCC in comparison to the TME tissues was observed. The expression of TGFβ3 mRNA in RCC was significantly lower than in the TME tissue and the expression in the TME was significantly higher comparing to the NK tissues (Fig. 1).

**Table 2:** The expression (mRNA) of genes of the TGFβ/Smads pathway in renal cell carcinoma (RCC), tumor microenvironment (TME) and in normal kidney (NK) tissues

Name of genes of tgfβ system	Renal cell carcinoma (RCC)	Tumor microenvironment (TME)	Normal kidney (NK)	*P* value		
	RQ median	RQ median	RQ median	RCC *vs* TME	RCC *vs* NK	TME *vs* NK
	Q_L_-Q_U_	Q_L_-Q_U_	Q_L_-Q_U_			
TGFβ1	0.32369	0.43653	0.055685	0.955	0.054	0.045*
	0.12143–1.0209	0.081821–0.987	0.005129–0.40509			
TGFβ2	0.024995	0.74453	0.26847	0.001*	0.066	0.252
	0.0032959–0.03601	0.29152–1.004	0.055413–0.93522			
TGFβ3	0.22745	0.76097	0.36275	0.003*	0.451	0.049*
	0.07837–0.42001	0.39315–1.6883	0.073039–0.74596			
TGFβRI	0.010752	0.13719	0.1764	0.003*	0.017*	0.767
	0.0035252–0.035481	0.027563–0.78513	0.017871–0.68016			
TGFβRII	0.27283	0.67665	0.47762	0.189	0.256	0.601
	0.077826–0.83574	0.22149–1.3432	0.24779–0.9086			
TGFβRIII	0.037153	0.23571	0.40222	0.038*	0.01*	0.54
	0.014092–0.085095	0.021491–0.42179	0.05196–0.62464			
Smad1	0.14883	0.70739	0.74039	0.042*	0.109	0.54
	0.071443–0.5334	0.21478–1.1522	0.11858–0.99517			
Smad2	0.0063883	0.39962	0.35573	0.038*	0.042*	1.0
	0.000029–0.03537	0.041941–0.58169	0.020798–0.70812			
Smad3	0.13378	0.38919	0.56256	0.197*	0.062	0.649
	0.06484–0.22239	0.068861–0.75292	0.10551–0.76015			
Smad4	0.0727	0.22957	0.35159	0.065	0.089	0.867
	0.025388–0.14947	0.03054–1.0494	0.037582–0.84208			
Smad5	0.041387	0.10429	0.29191	0.223	0.094	0.535
	0.017457–0.11984	0.028104–0.87876	0.020643–0.71663			
Smad6	0.18543	0.47185	0,42,403	0.018*	0.062	0.423
	0.031282–0.33276	0.20094–1.1071	0.096253–0.89213			
Smad7	0.15792	0.39406	0.22378	0.281	0.85	0.29
	0.064938–0.42322	0.12337–0.65216	0.080014–0.4973			

*Indicates statistical significance *p* < 0.05

*RQ* relative quantification, relative gene expression levels, *Q*_*L*_ lower quartile, *Q*_*U*_ upper quartile, *TGFβ1-3 *transforming growth factor 1–3, *TGFβRI-III *TGFβ type I-III receptors

**Fig. 2:**
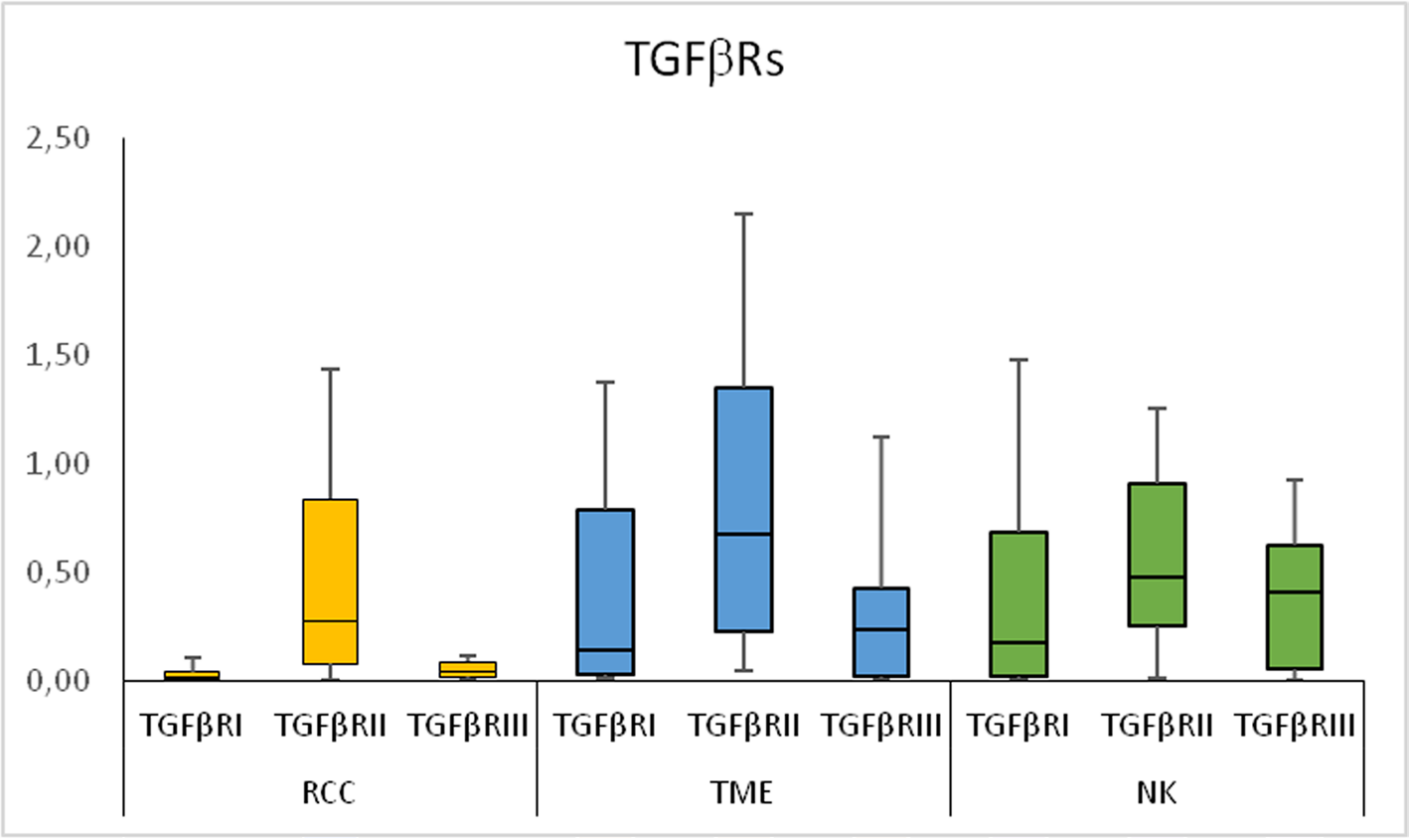
The expression (mRNA) of genes of the transforming growth factors receptors (TGFβRs) in renal cell carcinoma (RCC), tumor microenvironment (TME) and in normal kidney (NK) tissues. *RQ* relative quantification, Relative gene expression levels, median, *Q*_*L*_ lower quartile, *Q*_*U*_ upper quartile, lowest value, highest value are presented

The expression levels of TGFβRI and TGFβRIII mRNA in RCC were significantly lower than those in the TME and the NK tissues, but they did not differ between the TME and the NK tissues. There was no significant difference in the expression levels of TGFβRII mRNA between RCC, the TME, and the NK tissues (Fig. 2).

**Fig. 3:**
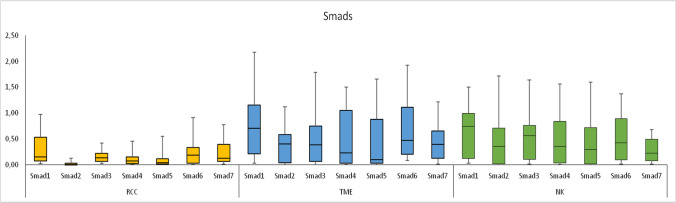
The expression (mRNA) of genes of the Smads in renal cell carcinoma (RCC), tumor microenvironment (TME) and in normal kidney (NK) tissues. *RQ* relative quantification, Relative gene expression levels, median, *Q*_*L*_ lower quartile, *Q*_*U*_ upper quartile, lowest value, highest value are presented

The expression levels of Smad1 and Smad3 mRNA were significantly lower in RCC than in the TME tissues, but it did not differ between RCC and the NK tissues. The expression of Smad2 mRNA in RCC was significantly lower than those in the TME and the NK tissues. However, there were no significant differences in the expression levels of Smad4, and Smad5 mRNA between RCC, the TME, and the NK tissues (Fig. 3).

The expression levels of Smad6 mRNA were significantly lower in RCC than in the TME tissue. However, there was no significant difference in the expression level of Smad7 mRNA between RCC, the TME, and the NK tissues (Fig. 3).

No significant differences in the expression of genes of the TGFβ signaling pathway components (receptors and signal proteins) were observed between the TME and the NK tissues (Fig. 3).

The description of the correlations found within the TGFβ system (Table 3, Supplementary Table 3a—online resource) can be found in the Discussion section. Among them, a positive correlation between the age of patients and the expression of Smad6 mRNA in the KC tumor (*n* = 33; R 0.509, *p* 0.013), including RCC (*n* = 29; R 0.521, *p* 0.019) was also found.

**Table 3:**
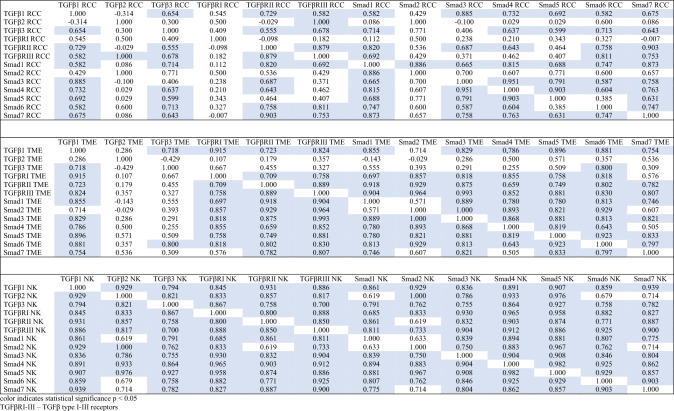
Correlation coefficients among the expressions (mRNA) of genes of the TGFβ/Smads pathway in renal cell carcinoma (RCC), tumor microenvironment (TME) and normal kidney (NK) tissues

## Discussion

**Fig. 4:**
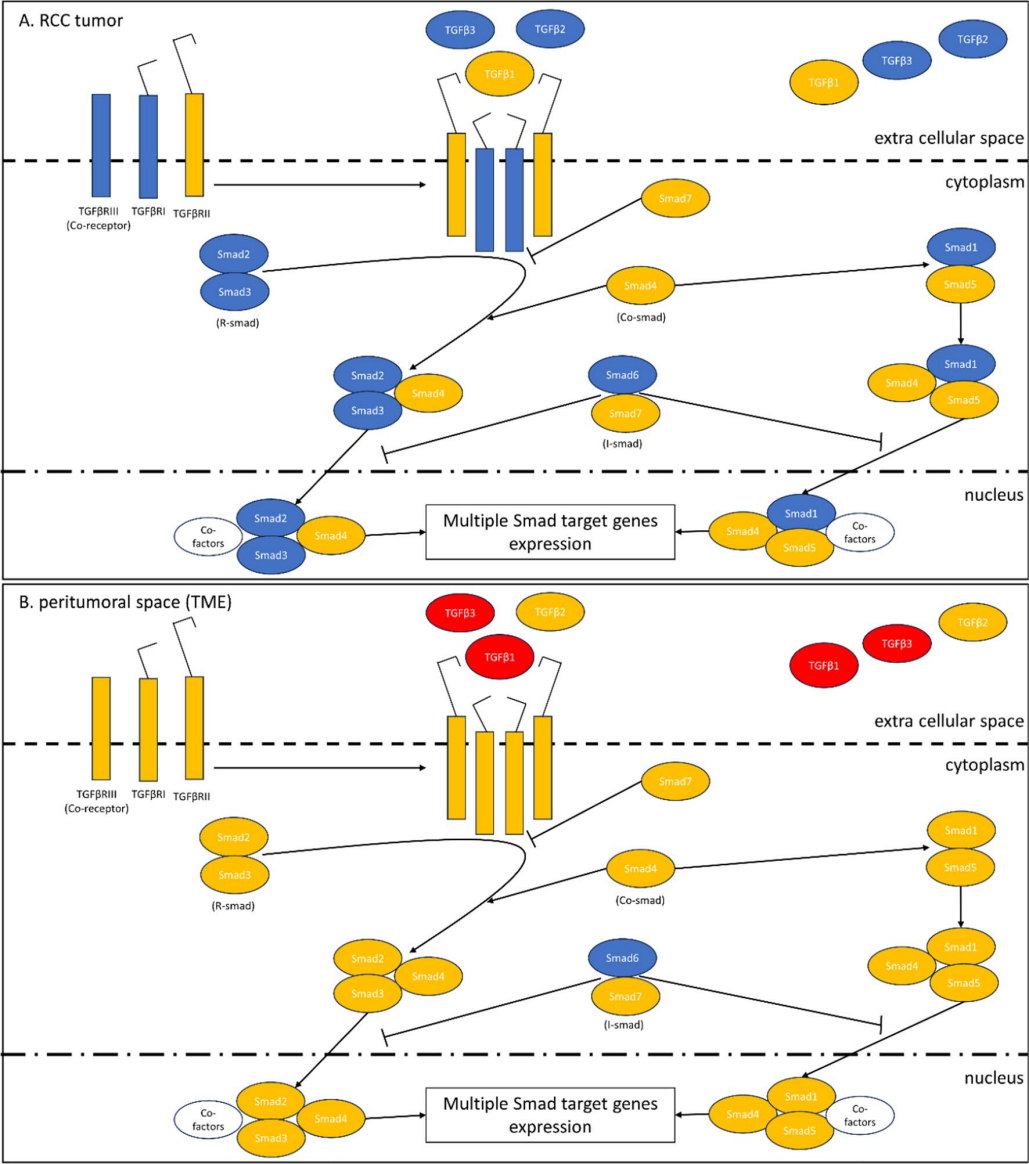
Diagram showing the possible TGFβ/Smads pathway in **A** renal cell carcinoma (RCC) and **B** tumor microenvironment (TME) tissues. Red color indicates overexpression of genes (mRNA); Blue color indicates underexpression of genes (mRNA). Transforming growth factors 1–3 (TGFβ1-3) act through transmembrane TGFβ type I-III receptors (TGFβRI-III) associated with the Smad signal transducer system. The TGFβs bind to the heterotetrameric TGFβ receptor complex, consisting of TGFβRI and TGFβRII dimers. TGFβRIII is a co-receptor presenting TGFβs to the TGFβRI. After being activated by phosphorylation, the receptor complex transmits the signal onto intracellular proteins Smad2 and Smad3 (R-Smad). As a result, R-Smad dissociates from the TGFβ-receptor and forms a complex with Co-Smad i.e., Smad4. The newly formed complex is then transported into cell nucleus, where it regulates the transcription of TGFβ-dependent genes. The signal transduction can be inhibited by Smad6 and Smad7 (i. e., I-Smads). Smad7 forms a stable complex with activated TGFβRI, thereby impairing the R-Smad phosphorylation. This, in result, inhibits the whole signal cascade. The signaling pathway of Smad1 and Smad5 is stimulated by a BMP receptor, and is connected to the above cascade through Smad4. The underexpression of genes of the TGFβ/Smads pathway inside a malignant tumor may result in loss of the antiproliferative and pro-apoptotic activity of this cytokine. The overexpression of the TGFβ1 i TGFβ3 genes in the TME does not coincide with downregulation of their receptors. At the same time, the low Smad6 expression, together with high expression of TGFβs, and unaffected expression of other Smads, indicates a shift towards signal induction and transmission. The overexpression of the TGFβs genes in the TME may result in an immunosuppressive effect in the peritumoral space and may have an antiproliferative and pro-apoptotic effect on non-neoplastic cells present within the TME

This article, in essence, examines the role of growth factors in neoplastic transformation, immunological processes, and angiogenesis taking place during organ remodeling [1, 4–6, 29–32]. The transcriptomic analysis of the TGFβ system in patients with the KC is in line with the contemporary research trends in oncology, urology, and immunology. Summing up the results of the study, the expression of genes of the TGFβ/Smads pathway becomes dysregulated within the RCC tissues, as well as in the peritumoral space (TME). Expression of TGFβ1, TGFβ3 in the TME is increased in comparison to the NK tissues. Expression of TGFβ2, TGFβ3, TGFβRI, TGFβRIII, Smad1, Smad2, Smad3, Smad6 is lower in RCC in comparison to the TME tissues. TGFβRI, TGFβRIII, Smad2 in RCC are underexpressed also comparing to the NK tissues (Table 2, Figs. 1, 2, 3, 4). The underexpression of genes of the TGFβ/Smads pathway inside a malignant tumor may result in loss of the antiproliferative and pro-apoptotic activity of this cytokine. The overexpression of the TGFβs genes in the TME comparing to the NK, as well as the TGFβ/Smads pathway genes in the TME comparing to the tumor, may result in an immunosuppressive effect in the space surrounding the tumor and may have an antiproliferative and pro-apoptotic effect on non-neoplastic cells present within the TME. We were particularly interested in the activity of the TGFβ system in the tissues adjacent to the tumor, as we believe that the functional and morphological consistency of this space may determine the aggressiveness of the tumor and the time in which the neoplastic process spreads. The observations made on the subcellular and cellular levels do not necessarily translate into the activity of the TGFβ system on the autocrine, paracrine, or endocrine levels, but there is no doubt that they precede changes on higher levels of the organization of the diseased tissue and the whole organism. According to the literature, the mRNA expression of TGFβ1 in ccRCC was significantly higher than in normal tissues [33]. In our study, we observed this in the TME of RCC (Table 2). There were no significant differences in the expression levels of TGFβ receptors (TGFβRI-II) and Smads (Smad2, Smad3, Smad4) mRNAs between ccRCC and the corresponding NK tissues. However, decreased expression of TGFβRIII was observed in ccRCC tissues [26]. Our observations regarding the comparison of RCC and the NK tissues are partially similar (Table 2), but we found no reports comparing TGFβ/Smads pathway transcripts or proteins between the TME and the tumor, or NK tissue. We have also observed changes in the TGFβ system gene expression in other pathophysiological situations. In thyroid tissues, strict regulation of transcriptional activity of TGFβ1 and their receptors TGFβRI-III genes observed in normal tissues is completely disturbed in papillary thyroid cancer (PTC). In PTC tissue, higher transcriptional activity of TGFβ1 gene and lower transcriptional activity of TGFβRII and TGFβRIII genes in comparison with benign tissues suggests its importance of this cytokine and its receptors in pathogenesis of cancer development [5] and are essentially similar to our observations in patients with RCC.

Feedback loops play a pivotal role in regulation of TGFβ signaling in normal conditions. It is however not fully understood, how dysregulation of this system contributes to pathogenesis of diseases [34]. TGFβ/Smad pathway contains the negative feedback loop mediated through Smad7 competitive binding to TGFβRI which results in blocking of the TGFβ/Smad pathway signaling [13]. Is this mechanism altered in RCC? Analysis of the correlations between the elements of the TGFβ system might provide some insight (Table 3, Supplementary Table 3a—online resource). Did it surprise us that all the found correlations between expressions of all TGFβ system genes were positive? Only that no negative correlation was found between Smad6/Smad7 and Smad4. However, on the subcellular and cellular level, such interactions are rather short-lived and pulsatile, as opposed to long-term interactions in negative feedback loops on the endocrine level. Therefore, it is not surprising that there is a positive correlation between I-Smads and Co-Smad on the intracellular level. Analysis of the expression of all genes of the TGFβ system indicates comprehensive and consistent activity of the TGFβ/Smads pathway in the healthy tissue—out of the 78 theoretically possible correlations, only 6 were not found. Such consistency in the functioning of the TGFβ system is disrupted in both the TME and the cancer tissues, resulting in malfunctioning of the system, especially within the tumor—out of the 78 possible correlations, 26 were not found in TME, and 37 in the RCC tissue. Narrowing the correlation analysis to TGFβ1/TGFRI/Smad cascade (the best known genes of the TGFβ system), it can be seen that in healthy tissue TGFβ1 and TGFβRI expression correlate with expression of other genes of the system, while Smad7 expression does not positively correlate with Smad2. In RCC tissue, TGFβ1 expression does not positively correlate with TGFβRI and Smad 2, while TGFβRI expression does not correlate with all Smads (indicating a disruption of the signaling pathway). In the TME, TGFβ1 expression does not positively correlate with Smad2 expression (indicating disruption of the signaling pathway), TGFβRI expression does not positively correlate with Smad7 expression (which may be a response to this signal disruption), and Smad7 expression does not positively correlate with Smad2 expression (which may also be a response to the above-mentioned disruption). Moreover, keeping in mind the inhibitory effect of Smad6 on the activity of the TGFβ system, it could be discussed, whether the observed positive correlation between the age of KC patients and the expression of Smad6 mRNA in tumor contribute to a slower course of cancer disease in the elderly.

According to the TCGA database, in the primary RCC tumor samples TGFβ1 so far was analyzed in 71 patients (1 mutation was detected), TGFβ2 in 167 cases (1 mutation), TGFβ3 in 263 cases (1 mutation), TGFβRI in 154 cases (1 mutation), TGFβRII in 536 cases (1 mutation), Smad1 in 117 cases (2 mutations), Smad2 in 168 cases (4 mutations), Smad3 in 77 cases (1 mutations), Smad4 in 166 cases (1 mutations), Smad5 in 340 cases, Smad6 in 78 cases (1 mutation), Smad7 in 165 cases (2 mutations), Smad9 in 164 cases (2 mutations were detected). The TCGA transcriptomic data for TGFβ signaling pathway genes in RCC are also available [28]. In addition, analysis of TCGA data by the use of the Tumor Immune Estimation Resource (TIMER) (https://cistrome.shinyapps.io/timer/) shows that in various types of cancer gene expression may correlate with the amount of immune infiltrates. TIMER is a web tool for a comprehensive analysis of the complex interaction of immune cells (B cells, CD4 + T cells, CD8 + T cells, neutrophils, macrophages, and dendritic cells) in tumors and normal tissues. Its statistical method is validated by the use of pathological estimations [35–37]. Transcripts of specific genes, including the TGFβ system genes, in combination with immunocompetent cells, can create a distinctive abnormal microenvironment, both inside the tumor and in its surroundings (TME). Possibly in the future, TCGA and TIMER, absorbing more and more data, will also include the TME in their analyses.

The duality of TGFβ/Smad pathway roles have been demonstrated in tumorigenesis. TGFβ was originally identified as an antitumor cytokine [13, 38]. Some studies have shown that TGFβ can induce apoptosis in RCC, and c-Ski (a transcriptional corepressor of Smad) signaling can weaken the antitumor effect of TGFβ by inhibiting TGFβ signal transduction [26]. Disruption of the TGFβ signaling pathway inside the cancer cells may be related to the promotion of the tumor [24, 39]. Also, there is increasing evidence that it plays an important role in the TME in facilitating cancer progression [13, 38]. TGFβ actively shapes the TME via modulating the host immunity. TGFβ is produced not only by cancer cells but also by different types of immune cells within the TME [13]. TGFβ drives cancer immune evasion in part by inducing Treg and limiting CD8 + T cell function [40]. TGFβ1 is also able to promote migration and invasion of RCC cells [41].

For the treatment of cancer, numerous promising immunotherapy approaches have been emerged by targeting TME [42]. Immune checkpoint blockade targeting programmed cell death protein 1 (PD-1) or its ligand PD-L1 is one of effective first-line approach in cancer immunotherapy. However, most patients fail to respond clinically. One potential reason is the accumulation of immunosuppressive TGFβ in TME [2, 40]. TGFβ attenuates tumor response to PD-L1 blockade by contributing to exclusion of T cells. Combination of TGFβ inhibition and immunotherapy induces complete responses in mouse models [43, 44]. Glycoprotein-A repetitions predominant (GARP) is a cell surface docking receptor for activating latent TGFβ1, TGFβ2, and TGFβ3, with its expression restricted predominantly to effector Treg and cancer cells [2, 40]. Selective targeting of GARP-latentTGFβ axis in TME augments PD-1 blockade via enhancing CD8 + T cell antitumor immunity [40]. Selective inhibition of TGFβ1 activation overcomes primary resistance to ICB therapy by altering tumor immune landscape [45]. Blockade of the TGFβ signaling has shown promising prospects in cancer therapy [21, 38, 43, 46], due to attenuation of the Treg-mediated immunosuppression, increase in the T cell cytotoxicity, facilitating of the T cell penetration into the center of the tumor, as well as inhibition of epithelial-mesenchymal transition, resulting in vigorous anti-tumor immunity and tumor regression [21]. Since, the activation of TGFβ signaling impairs the antitumor activity of cytotoxic T cells, and the suppression of TGFβ promotes the anticancer immune response against cancer cells [22], is blocking of TGFβ signaling in the peritumoral space likely to effectively disrupt the progression of RCC? The results of our study suggest that a possible blockade of TGFβ signaling could be used, but rather in the in space surrounding the tumor than in the tumor itself. Despite the underexpression of TGFβ signaling pathway inside the tumor, the systemic application of TGFβ blockade in patients before and after RCC surgery could also be justified—in order to disrupt the tumor-induced immunosuppression.

### Supplementary Information

Below is the link to the electronic supplementary material.

Supplementary file1 Supplementary Table 3a p value for correlation coefficients (presented in Table 3) among the expressions (mRNA) of genes of the TGFβ/Smads pathway in renal cell carcinoma (RCC), tumor microenvironment (TME) and in normal kidney (NK) tissues (online resource) (PDF 200 KB)

## Data Availability

The data that support the findings of this study are available from the corresponding author upon reasonable request.

## Abbreviations

ccRCC: Clear cell renal cell carcinoma
Co-Smad: Common partner Smad
I-Smad: Inhibitory Smads
KC: Kidney cancer
RCC: Renal cell carcinoma
R-Smad: Receptor regulated Smads
TGFβ: Transforming growth factor β
TGFβRI-III: TGFβ type I-III receptors
TME: Tumor microenvironment
Treg: Regulatory T cells
