# Supplementary material for: Transforming growth factors β and their signaling pathway in renal cell carcinoma and peritumoral space—transcriptome analysis
Source: Clin Transl Oncol. 2023 Dec 12;26(5):1229–39. doi: 10.1007/s12094-023-03350-y (PMC11026247; doi:10.1007/s12094-023-03350-y)
Supplement: Supplementary file 1 — Supplementary file1 Supplementary Table 3a p value for correlation coefficients (presented in Table 3) among the expressions (mRNA) of genes of the TGFβ/Smads pathway in renal cell carcinoma (RCC), tumor microenvironment (TME) and in normal kidney (NK) tissues (online resource) (PDF 200 KB) [file 12094_2023_3350_MOESM1_ESM.pdf]

Transforming Growth Factors β and their signaling pathway in renal cell carcinoma and peritumoral space - transcriptome analysis

Supplementary Table 3a *p* value for correlation coefficients (presented in Table 3) among the expressions (mRNA) of genes of the TGFβ/Smads pathway in renal cell carcinoma (RCC), tumor microenvironment (TME) and in normal kidney (NK) tissues

	TGFβ1 RCC	TGFβ2 RCC	TGFβ3 RCC	TGFβRI RCC	TGFβRII RCC	TGFβRIII RCC	Smad1 RCC	Smad2 RCC	Smad3 RCC	Smad4 RCC	Smad5 RCC	Smad6 RCC	Smad7 RCC
TGFβ1 RCC	1.000	0.564	0.015	0.067	0.001	0.037	0.029	0.419	0.000	0.003	0.006	0.037	0.006
TGFβ2 RCC	0.564	1.000	0.683	0.450	1.000	0.017	0.919	0.083	0.950	1.000	1.000	0.242	0.919
TGFβ3 RCC	0.015	0.683	1.000	0.212	0.049	0.015	0.006	0.103	0.191	0.019	0.031	0.009	0.018
TGFβRI RCC	0.067	0.450	0.212	1.000	0.762	0.593	0.729	0.450	0.457	0.513	0.276	0.326	0.983
TGFβRII RCC	0.001	1.000	0.049	0.762	1.000	0.000	0.000	0.236	0.010	0.010	0.095	0.003	0.000
TGFβRIII RCC	0.037	0.017	0.015	0.593	0.000	1.000	0.009	0.419	0.236	0.112	0.168	0.001	0.003
Smad1 RCC	0.029	0.919	0.006	0.729	0.000	0.009	1.000	0.033	0.013	0.000	0.007	0.003	0.000
Smad2 RCC	0.419	0.083	0.103	0.450	0.236	0.419	0.033	1.000	0.233	0.167	0.103	0.350	0.175
Smad3 RCC	0.000	0.950	0.191	0.457	0.010	0.236	0.013	0.233	1.000	0.000	0.001	0.045	0.003
Smad4 RCC	0.003	1.000	0.019	0.513	0.010	0.112	0.000	0.167	0.000	1.000	0.000	0.029	0.002
Smad5 RCC	0.006	1.000	0.031	0.276	0.095	0.168	0.007	0.103	0.001	0.000	1.000	0.194	0.016
Smad6 RCC	0.037	0.242	0.009	0.326	0.003	0.001	0.003	0.350	0.045	0.029	0.194	1.000	0.003
Smad7 RCC	0.006	0.919	0.018	0.983	0.000	0.003	0.000	0.175	0.003	0.002	0.016	0.003	1.000

	TGFβ1 TME	TGFβ2 TME	TGFβ3 TME	TGFβRI TME	TGFβRII TME	TGFβRIII TME	Smad1 TME	Smad2 TME	Smad3 TME	Smad4 TME	Smad5 TME	Smad6 TME	Smad7 TME
TGFβ1 TME	1.000	0.556	0.013	0.000	0.003	0.000	0.000	0.088	0.000	0.001	0.000	0.000	0.002
TGFβ2 TME	0.556	1.000	0.419	0.840	0.713	0.444	0.783	1.000	0.556	0.267	0.200	0.444	0.236
TGFβ3 TME	0.013	0.419	1.000	0.059	0.160	0.326	0.077	0.396	0.385	0.450	0.110	0.003	0.355
TGFβRI TME	0.000	0.840	0.059	1.000	0.022	0.011	0.025	0.024	0.004	0.002	0.011	0.004	0.082
TGFβRII TME	0.003	0.713	0.160	0.022	1.000	0.000	0.000	0.007	0.000	0.014	0.002	0.001	0.001
TGFβRIII TME	0.000	0.444	0.326	0.011	0.000	1.000	0.000	0.003	0.000	0.000	0.000	0.000	0.000
Smad1 TME	0.000	0.783	0.077	0.025	0.000	0.000	1.000	0.200	0.000	0.002	0.001	0.001	0.001
Smad2 TME	0.088	1.000	0.396	0.024	0.007	0.003	0.200	1.000	0.000	0.012	0.034	0.007	0.167
Smad3 TME	0.000	0.556	0.385	0.004	0.000	0.000	0.000	0.000	1.000	0.000	0.000	0.001	0.000
Smad4 TME	0.001	0.267	0.450	0.002	0.014	0.000	0.002	0.012	0.000	1.000	0.001	0.024	0.078
Smad5 TME	0.000	0.200	0.110	0.011	0.002	0.000	0.001	0.034	0.000	0.001	1.000	0.000	0.000
Smad6 TME	0.000	0.444	0.003	0.004	0.001	0.000	0.001	0.007	0.001	0.024	0.000	1.000	0.001
Smad7 TME	0.002	0.236	0.355	0.082	0.001	0.000	0.001	0.167	0.000	0.078	0.000	0.001	1.000

	TGFβ1 NK	TGFβ2 NK	TGFβ3 NK	TGFβRI NK	TGFβRII NK	TGFβRIII NK	Smad1 NK	Smad2 NK	Smad3 NK	Smad4 NK	Smad5 NK	Smad6 NK	Smad7 NK
TGFβ1 NK	1.000	0.002	0.006	0.001	0.000	0.000	0.000	0.002	0.000	0.000	0.000	0.000	0.000
TGFβ2 NK	0.002	1.000	0.034	0.015	0.011	0.011	0.115	0.000	0.028	0.001	0.000	0.110	0.058
TGFβ3 NK	0.006	0.034	1.000	0.001	0.011	0.016	0.004	0.037	0.007	0.001	0.000	0.011	0.008
TGFβRI NK	0.001	0.015	0.001	1.000	0.003	0.000	0.014	0.008	0.000	0.000	0.000	0.000	0.002
TGFβRII NK	0.000	0.011	0.011	0.003	1.000	0.000	0.000	0.115	0.000	0.000	0.000	0.001	0.000
TGFβRIII NK	0.000	0.011	0.016	0.000	0.000	1.000	0.000	0.031	0.000	0.000	0.000	0.000	0.000
Smad1 NK	0.000	0.115	0.004	0.014	0.000	0.000	1.000	0.076	0.000	0.000	0.000	0.000	0.001
Smad2 NK	0.002	0.000	0.037	0.008	0.115	0.031	0.076	1.000	0.025	0.003	0.000	0.037	0.058
Smad3 NK	0.000	0.028	0.007	0.000	0.000	0.000	0.000	0.025	1.000	0.000	0.000	0.000	0.000
Smad4 NK	0.000	0.001	0.001	0.000	0.000	0.000	0.000	0.003	0.000	1.000	0.000	0.000	0.000
Smad5 NK	0.000	0.000	0.000	0.000	0.000	0.000	0.000	0.000	0.000	0.000	1.000	0.000	0.000
Smad6 NK	0.000	0.110	0.011	0.000	0.001	0.000	0.000	0.037	0.000	0.000	0.000	1.000	0.000
Smad7 NK	0.000	0.058	0.008	0.002	0.000	0.000	0.001	0.058	0.000	0.000	0.000	0.000	1.000

color indicates statistical significance $p < 0.05$; TGFβRI-III – TGFβ type I-III receptors

*Dariusz Kajdaniuk, Dorota Hudy, Joanna Katarzyna Strzelczyk, Krystyna Młynarek, Szymon Słomian, Andrzej Potyka, Ewa Szymonik, Janusz Strzelczyk, Wanda Foltyn, Beata Kos-Kudła, Bogdan Marek
*corresponding author: Department of Pathophysiology,
Chair of Pathophysiology and Endocrinology,
Medical University of Silesia, Katowice
H. Jordana 19, 41-808 Zabrze, Poland
e-mail: dkajdaniuk@sum.edu.pl
